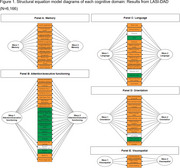# Risk and protective factors of cognitive decline in older adults from a nationally representative sample in India: Results from the LASI‐DAD

**DOI:** 10.1002/alz70860_099409

**Published:** 2025-12-23

**Authors:** Alden L. Gross

**Affiliations:** ^1^ Johns Hopkins Bloomberg School of Public Health, Baltimore, MD, USA

## Abstract

**Background:**

Many studies have proposed important risk and protective factors for dementia, although most are from Western samples in high‐income settings, including those summarized in the 2024 Lancet Commission on dementia. Our goal was to characterize modifiable risk factors of cognitive decline in a nationally representative study of older adults in India.

**Method:**

We used a detailed neuropsychological battery administered to *N* = 6,166 community‐living adults aged 60+ years across two waves of the nationally‐representative Harmonized Diagnostic Assessment of Dementia for the Longitudinal Aging Study in India (LASI‐DAD). We established the factor structure of the cognitive battery, derived co‐calibrated measures of cognitive functioning across waves, and evaluated associations of modifiable and non‐modifiable risk factors for late‐life cognitive decline with up to 6.4 years of follow‐up. Risk factors included demographic characteristics, self‐reported and objective health characteristics (i.e., markers of cardiovascular disease), health behaviors, and sensory function.

**Result:**

Confirmatory factor analyses to co‐calibrate measures of general cognitive functioning, memory, executive functioning/attention, language/fluency, visuospatial ability, and orientation domains across LASI‐DAD waves 1 and 2 fit well to the data. Most risk factors, particularly demographics and cardiovascular characteristics, were associated with steeper cognitive decline.

**Conclusion:**

Most risk factors we evaluated were associated with decline in expected directions, highlighting the potential generalizability of previously identified risk factors for dementia in India. Summary measures of cognitive domains derived in this study can be used in future longitudinal research on cognitive aging in India.